# A Hybrid Gate Dielectrics of Ion Gel with Ultra-Thin Passivation Layer for High-Performance Transistors Based on Two-Dimensional Semiconductor Channels

**DOI:** 10.1038/s41598-017-14649-6

**Published:** 2017-10-27

**Authors:** Hyunjin Jo, Jeong-Hun Choi, Cheol-Min Hyun, Seung-Young Seo, Da Young Kim, Chang-Min Kim, Myoung-Jae Lee, Jung-Dae Kwon, Hyoung-Seok Moon, Se-Hun Kwon, Ji-Hoon Ahn

**Affiliations:** 1 Department of Electronic Material Engineering, Korea Maritime and Ocean University, 727 Taejong-ro, Yeongdo-gu, Busan, 49112 Republic of Korea; 2 Department of Material Science and Engineering, Pohang University of Science and Technology, 77 Cheongam-Ro, Pohang, 790-784 Republic of Korea; 3 School of Materials Science and Engineering, Pusan National University, 30 Jangjeon-Dong Geumjeong-Gu, Busan, 609-735 Republic of Korea; 4 Daegu Gyeongbuk Institute of Science and Technology (DGIST), 333 Techno Jungang Daero, Hyeonpung-Myeon, Dalseong-Gun, Daegu, 42988 Republic of Korea; 5 Department of Advanced Functional Thin Films, Surface Technology Division, Korea Institute of Materials Science, 797 Changwondaero, Sungsan-Gu, Changwon, Gyeongnam 51508 Republic of Korea; 6 Energy Plant R&D Group, Korea Institute of Industrial Technology (KITECH), 30 Gwahaksandan 1-ro 60beon-gil, Gangseo-gu, Busan, 46742 Republic of Korea

## Abstract

We propose a hybrid gate structure for ion gel dielectrics using an ultra-thin Al_2_O_3_ passivation layer for realizing high-performance devices based on electric-double-layer capacitors. Electric-double-layer transistors can be applied to practical devices with flexibility and transparency as well as research on the fundamental physical properties of channel materials; however, they suffer from inherent unwanted leakage currents between electrodes, especially for channel materials with low off-currents. Therefore, the Al_2_O_3_ passivation layer was introduced between the metal electrodes and ion gel film as a leakage current barrier; this simple approach effectively reduced the leakage current without capacitance degradation. In addition, we confirmed that a monolayer MoS_2_ transistor fabricated with the proposed hybrid gate dielectric exhibited remarkably enhanced device properties compared to a transistor using a normal ion gel gate dielectric. Our findings on a simple method to improve the leakage current properties of ion gels could be applied extensively to realize high-performance electric-double-layer transistors utilizing various channel materials.

## Introduction

Ionic liquids provide the advantage of very large capacitances by the formation of nanogap capacitors at solid–liquid interfaces^1,2^, and electric double-layer-transistors (EDLTs) using ionic liquids have received significant attention because they show ultra-high capacitance and potential applicability in both practical flexible and transparent devices and studies on the fundamental physical properties of channel materials, including carrier transport phenomena, superconductivity, and thermoelectricity^3–13^. Despite this broad applicability, ionic liquids are limited in application to practical devices because of fluidity-related instability; however, this problem can be solved by introducing an ion gel, comprising a mixture of an ionic liquid and a polymer network, which maintains both the mechanical flexibility of the polymer and the large specific capacitance of the ionic liquid^14–19^. As demonstrated by printable and photo-patternable ion gels, large-area device integration and the realization of practical applications of stretchable and flexible devices have become possible^20,21^.

Meanwhile, in recent years, thin-film transistors (TFT) based on two-dimensional (2D) materials, such as hexagonal metal dichalcogenides, have been extensively studied for their potential applicability in emerging electronic and optoelectronic devices^22–25^. 2D material-based TFTs using ion gels as gate dielectrics have been reported to exhibit superior electrical properties, such as low threshold voltages, high carrier mobilities, transparency, and flexibility^26–29^. Braga *et al*. realized ambipolar transistors fabricated using exfoliated WS_2_ flakes with ionic liquid and proposed a method to determine the bandgap size directly^30^; Zhang *et al*. demonstrated the formation and detection of field-induced *p-n* junctions in MoS_2_ EDLTs^31^. Thus, ion gels as gate dielectrics for 2D material-based devices have several advantages, but their practicality must be improved to realize high-performance devices. Electric double-layer dielectrics, including ion gels, exhibit high leakage current levels between the gate and source/drain electrodes compared to those in inorganic-based gate dielectric^32^. For channel materials with greater gate leakage currents than intrinsic off-current levels leakage not only degrades the on/off characteristics of the devices but also increases the threshold voltage. For example, the off-current level of previously reported monolayer MoS_2_ transistors was in the range of 10^**−**10^–10^**−**12^ A^33–36^, which is less than the gate leakage current level of several nano-amperes at the operating voltage in ion-gel-gated devices^32,37^. Most reported ion-gel-gated transistors with 2D materials exhibit off-current levels exceeding the nano-ampere range, which might be greater than the intrinsic properties of the channel materials; in such cases, the transfer curves are expressed on linear rather than logarithmic scales. Therefore, it is important to reduce the leakage current through ion-gel dielectrics between the gate and source/drain electrodes to achieve higher-performance devices, particularly for channel materials with low off-current levels.

In this study, we propose a hybrid gate dielectric structure composed of an ion-gel film and ultra-thin Al_2_O_3_ passivation layer deposited by atomic layer deposition (ALD) to improve the electrical properties of the ion-gel-based 2D transistor, because Al_2_O_3_ films exhibit good leakage current properties and the ultra-thin Al_2_O_3_ layer can act as a leakage current barrier without causing significant capacitance drops^38–40^. Moreover, it is known that the ultra-thin Al_2_O_3_ films formed by ALD exhibit flexibility and transparency^35,41^, and therefore the hybrid dielectric structure could be suitable for application to flexible and transparent devices. To optimize the structure of the hybrid dielectrics, the variations in the electrical properties of the Al_2_O_3_ layer-modified ion gel film were systemically investigated as a function of the thickness of the Al_2_O_3_ passivation layer. Finally, the optimized dielectric structure was applied to a monolayer MoS_2_ transistor and compared to a transistor using a normal ion-gel gate dielectric to confirm the effect of the proposed hybrid dielectrics on the transistor performance.

## Results and Discussion

**Figure 1:**
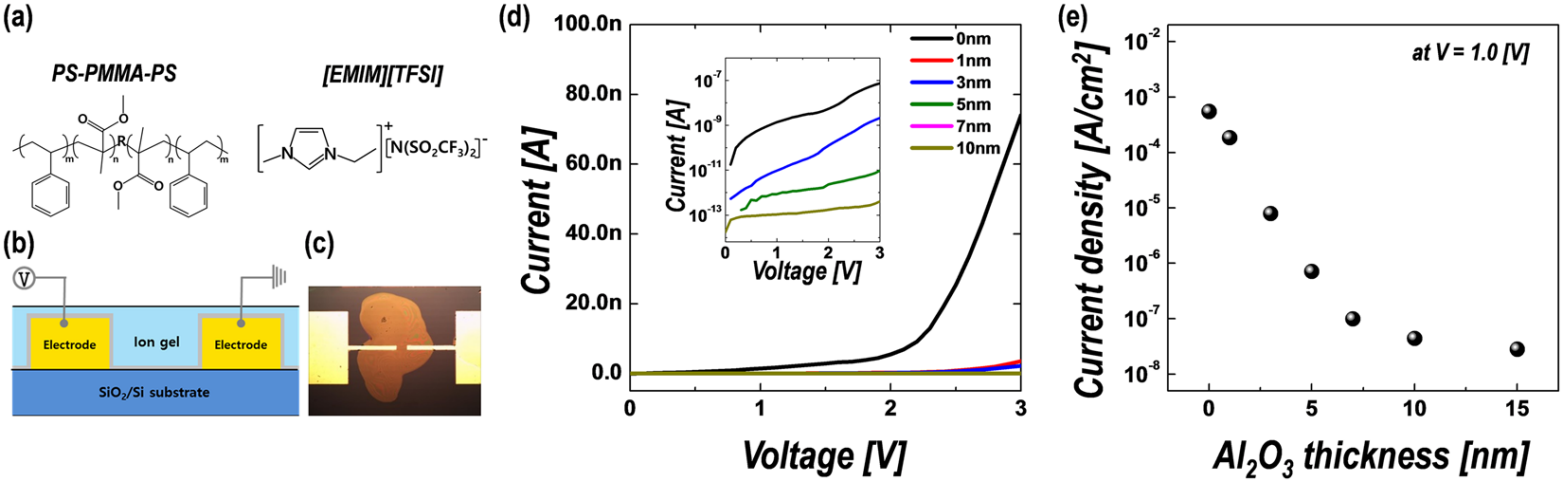
(**a**) Molecular structures of the triblock copolymer (PS-PMMA-PS) and ionic liquid ([EMIM][TFSI]) used for preparation of ion-gel film. (**b**) Schematic and (**c**) optical micrograph of the two-terminal device for characterization of electrical properties of hybrid dielectrics. (**d**) Current–voltage characteristics of hybrid dielectrics with various Al_2_O_3_ layer thicknesses. Inset: log-scale plot. (**e**) The variation of current density measured at 1 V as a function of Al_2_O_3_ layer thickness.

The ion gel films were prepared from a solution of the triblock copolymer poly(styrene-block-methyl methacrylate-block-styrene) (PS-PMMA-PS) and the ionic liquid 1-ethyl-3-methylimi dazolium bis(trifluoromethylsulfonyl)imide ([EMIM][TFSI]), in ethyl propionate as an organic solvent. The PS-PMMA-PS and [EMIM][TFSI], with molecular structures as shown in Fig. 1a, are commonly used base materials to fabricate ion gels for EDLTs^28,29^. First, we fabricated simple two-terminal devices, as shown in Fig. 1b,c, to investigate the effect of Al_2_O_3_ passivation on the electrical properties of the ion-gel films. The ultra-thin Al_2_O_3_ layers were deposited by thermal ALD using H_2_O as an oxidant at the temperature of 350 °C. The reasons for determining the deposition conditions of ALD-Al_2_O_3_ will be discussed later. Figure 1d shows the current (*I*)–voltage (*V*) characteristics of the two-terminal devices according to the thicknesses of the inserted Al_2_O_3_ layers between the metal electrodes and ion gels. With the insertion of the Al_2_O_3_ passivation layer, the leakage current is remarkably decreased. When the dielectric film comprises only the ion gel without a passivation layer, the current level at 1 V is ~10^**−**9^ A, indicating difficulty in accurately measuring the on/off characteristics for EDLTs devices with off-current levels below the nano-ampere scale. As shown in Fig. 1e and the inset of Fig. 1d, the current levels through the hybrid dielectric films exponentially decrease as the Al_2_O_3_ thickness is increased to 10 nm. This confirms that the leakage current can be effectively reduced by more than two and three orders of magnitude by inserting Al_2_O_3_ layers of only 3 and 5 nm in thickness, respectively.

**Figure 2:**
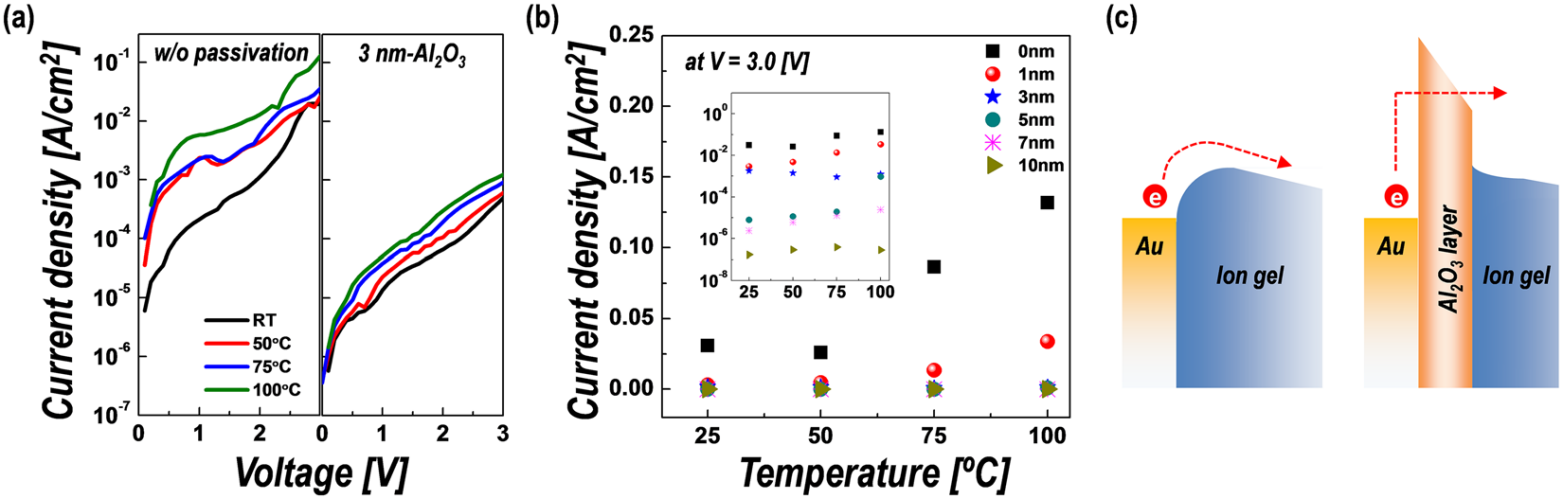
(**a**) *J–V* characteristics of ion gel film without passivation and with 3-nm-thick Al_2_O_3_ passivation layer, measured from room temperature to 100 °C. (**b**) Temperature-dependent current density of hybrid structure with various thicknesses of Al_2_O_3_ passivation layer, measured at 3 V bias. Inset: log-scale plot. (**c**) Schematics of current mechanism at metal–ion gel and metal–Al_2_O_3_–ion gel interfaces.

Furthermore, we investigated the temperature dependence of the leakage current through the hybrid ion-gel films to study the change of current mechanism caused by the insertion of the Al_2_O_3_ layer. Figure 2a shows the current density (*J*)–voltage (*V*) characteristics of the ion gel-based dielectric films without and with a 3-nm-thick Al_2_O_3_ passivation layer measured at the temperature range of room temperature to 100 °C. With the insertion of the ultra-thin Al_2_O_3_ layer, not only the overall leakage current level decreases, but also the temperature dependency of the current is decreased (data for samples with different passivation thicknesses is shown in Fig. S2 of the Supplementary Information). As indicated in Fig. 2b, the temperature dependency of the leakage current tends to decrease with increasing thickness of the Al_2_O_3_ passivation layer. Based on these observations, the current mechanism and the cause of the decrease in the leakage current by Al_2_O_3_ passivation can be explained as follows: In the absence of a passivation layer, the leakage current flows mainly by a thermionic mechanism, in which electrons flow over a specific barrier height between the metal electrode and the ion-gel dielectrics. However, the thin but large-bandgap (~8.9 eV) Al_2_O_3_ passivation layer located between the metal electrode and the ion gel acts as a leakage current barrier, preventing the flow of a direct thermionic current. Therefore, as the thickness of the passivation layer increases, the main current mechanism through the metal electrode to the ion gel-based dielectrics changes to electric field-induced tunneling, which is relatively insensitive to temperature, as indicated in the schematics in Fig. 2c. The results confirm that the leakage current characteristics are effectively improved by reducing the thermionic current in the dielectric structure with the placement of a large-bandgap Al_2_O_3_ passivation layer.

**Figure 3:**
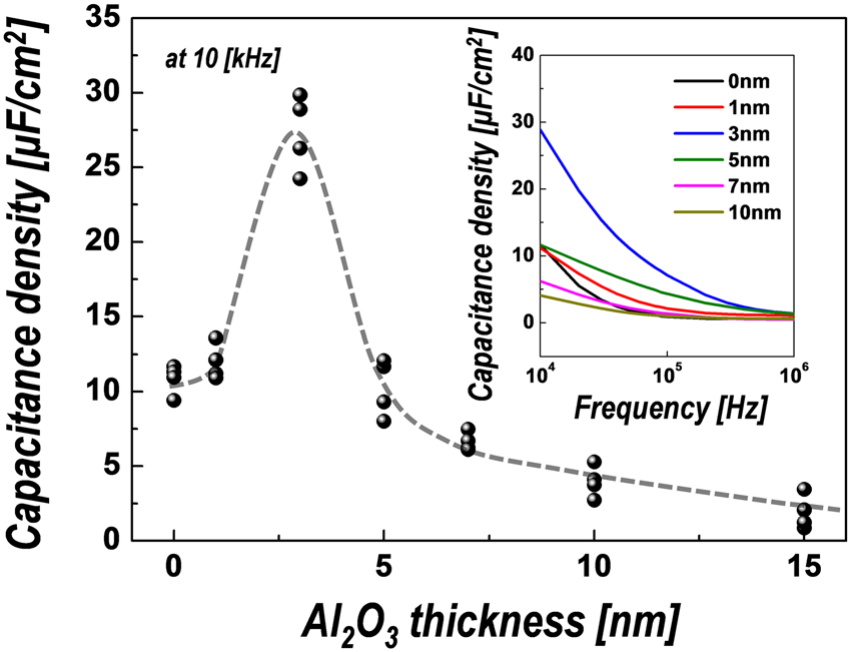
The variation of the capacitance density of two-terminal device with hybrid dielectrics depending on Al_2_O_3_ layer thickness with frequency of 10 kHz. Inset: frequency dependence of the capacitance for different Al_2_O_3_ thickness.

Meanwhile, although the leakage current characteristics of the device using a hybrid dielectric structure of ion gel with an Al_2_O_3_ passivation layer can be improved, the dielectric constant of Al_2_O_3_ is relatively low (~9), which can degrade the dielectric properties of the device. Therefore, we investigated the dielectric properties of ion gel-based hybrid dielectrics as a function of the thickness of the inserted Al_2_O_3_ layer. The capacitance density of the ion gel without passivation layer was ~10.8 μF/cm^**−**2^, which is comparable to those of previously reported gels (~5–10 μF/cm^**−**2^)^4,29,37^. The capacitance slightly increased by the insertion of an Al_2_O_3_ layer with the thickness of ~3 nm and then continuously decreased with further increases in the inserted Al_2_O_3_ thickness. The decrease in capacitance for relatively thick (>5 nm) Al_2_O_3_ layers can be explained by the relatively small dielectric constant of Al_2_O_3_ films, but it is difficult to understand the increase in capacitance for 3-nm-thick Al_2_O_3_-inserted ion-gel films. However, it was previously reported that self-discharge at the charged state in electric double-layer capacitors, caused by high leakage currents, could cause capacitance drops^42,43^. Therefore, the increase in capacitance for the sample with the 3-nm-thick Al_2_O_3_ layer was attributed to the decrease in leakage current by the insertion of the leakage current barrier. It is also observed that the frequency dependence of the capacitance tends to decrease with increases in the thickness of the Al_2_O_3_ passivation layer (see inset of Fig. 3). The most important point is that, although the low-dielectric-constant layer is inserted, the capacitance is not degraded until the Al_2_O_3_ thickness is increased to ~5 nm. Therefore, it can be expected that the hybrid gate structure of ion-gel dielectrics with ultra-thin Al_2_O_3_ passivation layers could effectively improve the transistor characteristics of EDLTs by reducing the gate leakage current without inducing capacitance drops.

**Figure 4:**
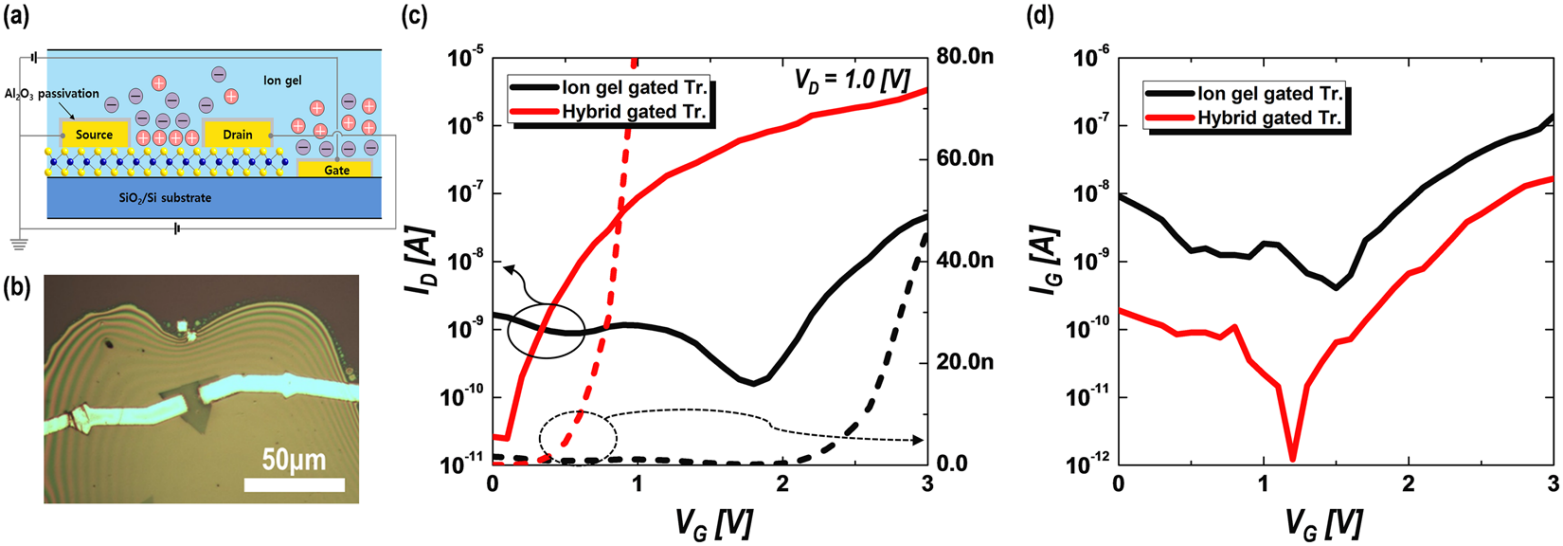
(**a**) Schematic and (**b**) optical micrograph of the hybrid-structure EDLTs fabricated using monolayer single-crystalline MoS_2_. (**c**) Transfer curve and (**d**) gate leakage current (absolute values) of the MoS_2_ EDLTs without passivation (ion gel gated Tr.) and with 3-nm-thick Al_2_O_3_ passivation layer (Hybrid gated Tr.) measured at drain voltage of 1.0 V under room temperature. Solid line and dashed line in transfer graph indicate the logarithmic-scale (left-side axis) and linear-scale (right-side axis) value of drain current, respectively.

Finally, the hybrid gate dielectric of ion-gel film with an ultra-thin Al_2_O_3_ passivation layer was applied to MoS_2_ EDLTs to confirm the effect of the proposed gate structure on the performance of the MoS_2_ transistors. Figure 4a,b show the schematic and optical micrograph of the fabricated MoS_2_ EDLT structure using the hybrid gate dielectric, respectively. Monolayer MoS_2_ crystals directly grown on a SiO_2_/Si substrate by chemical vapor deposition (CVD) were used as a transistor channel; source/drain and side gate electrodes were defined by the lift-off method. After formation of electrodes, 3-nm-thick Al_2_O_3_ layers were deposited by thermal ALD at 350 °C. ALD Al_2_O_3_ films are usually obtained using H_2_O, O_3_, or O_2_ plasma as the oxidant at the temperature range of 50 to 400 °C. In our case, we used H_2_O vapor as an oxidant to prevent damage to the MoS_2_ channel by ozone or O_2_ plasma. In addition, because ideal van der Waals 2D materials do not have dangling surface bonds, the precursor molecules for ALD growth were infrequently adsorbed on the 2D materials, so that thin film growth by ALD could be suppressed on the 2D materials. Therefore, the ALD-Al_2_O_3_ layer could be selectively deposited except on the 2D material surface without additional etching processes. However, the CVD-grown MoS_2_ could contain defect sites where the precursor can chemosorb; therefore, the ALD process for depositing the Al_2_O_3_ passivation layer was conducted at the temperature of 350 °C to minimize the chemisorption of precursors on the MoS_2_ surface^44^. After the deposition of the 3-nm-thick Al_2_O_3_ layer by ALD on the MoS_2_ crystals, it was confirmed that the Al_2_O_3_ layer was rarely deposited on the surface of the MoS_2_ crystals by atomic force microscopy analysis (see Fig. S3 of the Supplementary Information). In fact, since no degradation of capacitance was observed for inserted Al_2_O_3_ layer thicknesses below ~5 nm, it can be expected that even if the ultra-thin Al_2_O_3_ is partially deposited on MoS_2_, the transistor performance would not be negatively affected. The room-temperature transfer characteristics (*I*_*D*_ *–V*_*G*_) of the MoS_2_ EDLTs measured at the source–drain voltage (*V*_*D*_) of 1.0 V with and without Al_2_O_3_ passivation are indicated in Fig. 4c. For comparison, regardless of with and without passivation, the channel length and width were fixed at 10 and 10 μm, respectively. Whereas the on/off ratio of the ion gel-only gated transistor is ~10^2^ because of the relatively high off-current level of ~10^**−**9^ A, the on/off ratio of the hybrid-gated transistor is remarkably increased to ~10^5^. Moreover, it is observed that the threshold voltage is decreased by more than 2 V, and the subthreshold swing (SS) is also improved by inserting the ultra-thin Al_2_O_3_ layer between the electrodes. These improvements in the electrical properties of the transistor by the Al_2_O_3_ passivation layer are mainly attributed to the reduction of the leakage current between the gate and source/drain electrodes. By applying the hybrid gate structure of ion-gel dielectrics with 3-nm-thick Al_2_O_3_ passivation layers, as shown in Fig. 4d, the gate leakage current is significantly decreased by about two orders of magnitude (from several nano-amperes to several tens of pico-amperes at *I*_*D*_ = 1.0 V), which well corresponds with the off-current level of each transistor. In addition, the on-current level of the hybrid gated EDLTs was also greatly increased by Al_2_O_3_ passivation, which might be attributed to the reduced dielectric loss from the reduced gate leakage current.

## Conclusions

In this study, we systematically investigated the effect of inserting an Al_2_O_3_ passivation layer between the ion-gel dielectric film and metal electrode on the electrical properties of a transistor to propose a hybrid ion gel-based gate structure with improved properties. As the thickness of the inserted Al_2_O_3_ layer was increased, the leakage current through the ion gel-based dielectrics was exponentially decreased (more than two orders of magnitude even with an ultra-thin Al_2_O_3_ layer of 3 nm in thickness). Moreover, no degradation of capacitance was observed for inserted Al_2_O_3_ layer thicknesses below ~5 nm. The hybrid structured gate dielectric based on the ion-gel film with a 3-nm-thick Al_2_O_3_ layer was applied to a monolayer MoS_2_ transistor. The performance of the transistor was compared to that of another transistor using a normal ion-gel gate dielectric. Most transistor properties, such as the on/off ratio, threshold voltage, and subthreshold swing, were improved by the remarkable decrease of the gate leakage current. The proposed hybrid-structured gate dielectrics could be applied to versatile devices, especially with transparency and flexibility, based on EDLTs using various channel materials, as well as 2D material-based transistors.

## Methods

### Preparation of ion gel

The ion gel was prepared from a solution of a triblock copolymer, poly(styrene-block-methyl methacrylate-block-styrene) (PS-PMMA-PS, Polymer Source Inc.), and an ionic liquid, 1-ethyl-3-methylimi dazolium bis(trifluoromethylsulfonyl)imide ([EMIM][TFSI], Merck), in ethyl propionate as the organic solvent^28,29^. The polymer, ionic liquid, and solvent were mixed with the weight ratio of 0.5:9.5:20, and then the mixture was sonicated to obtain a homogeneous solution. This solution was drop-casted onto the metal electrodes of the device. Through annealing at 120 °C for 10 min, the solvent was evaporated and an ion gel film was formed through the physical association of the polymer blocks in the ionic liquid.

### Deposition of ultra-thin Al_2_O_3_ layer

To enhance the electrical properties of the gate dielectric based on the ion gel, an ultra-thin Al_2_O_3_ layer, deposited by ALD at 350 °C, was introduced between the metal electrode and ion-gel interface. Trimethylaluminum (TMA, Al(CH_3_)_3_) and water vapor (H_2_O) were used as precursor and oxidant, respectively; both canisters containing TMA and H_2_O were maintained at the temperature of 10 °C. The deposition process was performed under saturation conditions. One deposition cycle consisted of the exposure of TMA for 0.5 s, purging with N_2_ for 5 s, exposure of H_2_O for 0.5 s, and purging with N_2_ for 5 s.

### Device fabrication and characterization

To investigate the effect of Al_2_O_3_ passivation on the electrical properties, a simple two-terminal device was fabricated on SiO_2_/Si substrate. First, two electrodes were formed from 10-nm Ti/50-nm Au layers via a lift-off method using photolithography (Photo resist: AZ5214, AZ Electronic Materials) and e-beam evaporation. The hybrid gate dielectric was formed via the deposition of ultra-thin Al_2_O_3_ layers with thicknesses of 0–10 nm, followed by the drop-casting of the ion gel. Meanwhile, to apply the hybrid gate dielectric to field-effect transistor (FET) devices, MoS_2_ transistors were fabricated using monolayer crystals synthesized by CVD (see Fig. S1 of the Supplementary Information for more details on the synthesis process). Similar to the above method, source/drain and side gate electrodes were formed from 10-nm Ti/50-nm Au layers by a lift-off method. In this case, however, electron-beam lithography using poly(methyl methacrylate) (PMMA) was used instead of photolithography, because the source/drain electrodes required greater accuracy in formation on the MoS_2_ crystal. Finally, for comparison, two types of ion gel-based gate dielectrics were formed, one without passivation and the other with a 3-nm-thick Al_2_O_3_ passivation layer. After the device fabrication, capacitance–voltage (*C–V*), current–voltage (*I–V*), and FET transfer characteristics measurements were performed in a dark probe station using a semiconductor characterization system (Keithley, 4200-SCS).

## Electronic supplementary material

Supplementary Information
