# Supplementary material for: A Hybrid Gate Dielectrics of Ion Gel with Ultra-Thin Passivation Layer for High-Performance Transistors Based on Two-Dimensional Semiconductor Channels
Source: Sci Rep. 2017 Oct 27;7:14194. doi: 10.1038/s41598-017-14649-6 (PMC5660217; doi:10.1038/s41598-017-14649-6)
Supplement: Supplementary file 1 — Supplementary Information [file 41598_2017_14649_MOESM1_ESM.doc]

Supplementary Information

**A Hybrid Gate Dielectrics of Ion Gel with Ultra-Thin Passivation Layer for High-Performance Transistors Based on Two-Dimensional Semiconductor Channels**

Hyunjin Jo ^1, †^, Jeong-Hun Choi ^1, †^, Cheol-Min Hyun ^1, †^, Seung-Young Seo^2^, Da Young Kim^3^, Chang-Min Kim^3^, Myoung-Jae Lee^4^, Jung-Dae Kwon^5^, Hyoung-Seok Moon^6^, Se-Hun Kwon^3,*^ , and Ji-Hoon Ahn^1,*^

^1^Department of Electronic Material Engineering, Korea Maritime and Ocean University, 727 Taejong-ro, Yeongdo-gu, Busan 49112, Republic of Korea

^2^Department of Material Science and Engineering, Pohang University of Science and Technology, 77 Cheongam-Ro, Pohang 790-784, Republic of Korea

^3^School of Materials Science and Engineering, Pusan National University, 30 Jangjeon-Dong Geumjeong-Gu, Busan 609-735, Republic of Korea

^4^Daegu Gyeongbuk Institute of Science and Technology (DGIST), 333 Techno Jungang Daero, Hyeonpung-Myeon, Dalseong-Gun, Daegu 42988, Republic of Korea

^5^Department of Advanced Functional Thin Films, Surface Technology Division, Korea Institute of Materials Science, 797 Changwondaero, Sungsan-Gu, Changwon, Gyeongnam 51508, Republic of Korea

^6^Energy Plant R&D Group, Korea Institute of Industrial Technology (KITECH), 30 Gwahaksandan 1-ro 60beon-gil, Gangseo-gu, Busan 46742, Republic of Korea

^†^These authors contributed equally to this work

*Corresponding authors: Prof. Se-Hun Kwon (sehun@pusan.ac.kr) & Prof. Ji-Hoon Ahn (ajh1820@kmou.ac.kr)

**A. Monolayer MoS_2_ growth by CVD**


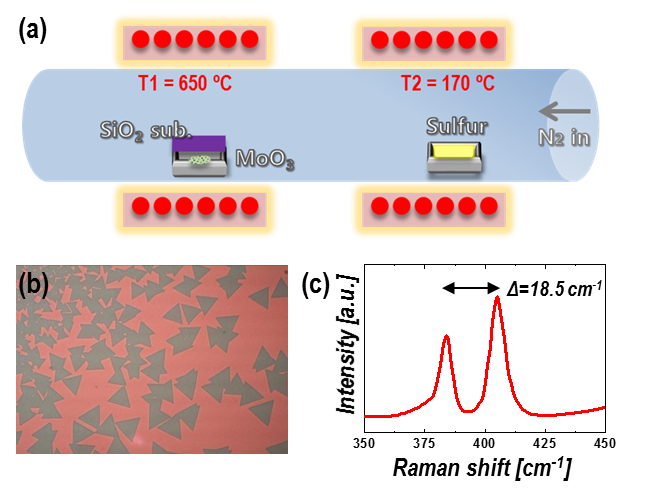


**Figure S1:** (a) Experimental scheme for the synthesis of MoS_2_ monolayer crystals. (b) Optical micrograph and (c) Raman spectrum of the MoS_2_ crystal.

Monolayer MoS_2_ crystals, used as the channel in the ion gel-based hybrid gated transistor, were synthesized on a SiO_2_/Si substrate by conventional thermal CVD in a two-zone furnace for accurate temperature control. Molybdenum trioxide (MoO_3_) powder and pure S powder were used as precursors. As shown in Figure S1a, the MoO_3_ powders and S powders, placed in separate Al_2_O_3_ boats, were loaded into the centers of the two heating zones of high and low temperatures (furnace 1 and 2, respectively), and the SiO_2_/Si substrate was placed face down and mounted on the top of the MoO_3_ boat. Prior to the growth process, the furnace was evacuated to 0.4 Pa and purged by flowing 100 sccm of N_2_ gas for 10 min. Then, the temperatures of furnace 1 and furnace 2 were gradually increased to 650 °C and 170 °C in 30 min, respectively, and maintained for 20 min for the MoS_2_ growth. In the cooling step, the chamber was cooled to 300 °C at 15 °C/min, before rapid cooling to room temperature.

Figure S1b shows the optical micrograph of triangular single crystals of the synthesized MoS_2_ of several tens of micrometers in width. We confirmed that the Raman spectra (Figure S1c) and the spacing between A and B peaks well corresponded to the previously reported value for monolayer MoS_2_ crystals.^1^

**B. *J-V* characteristics of hybrid ion-gel films**


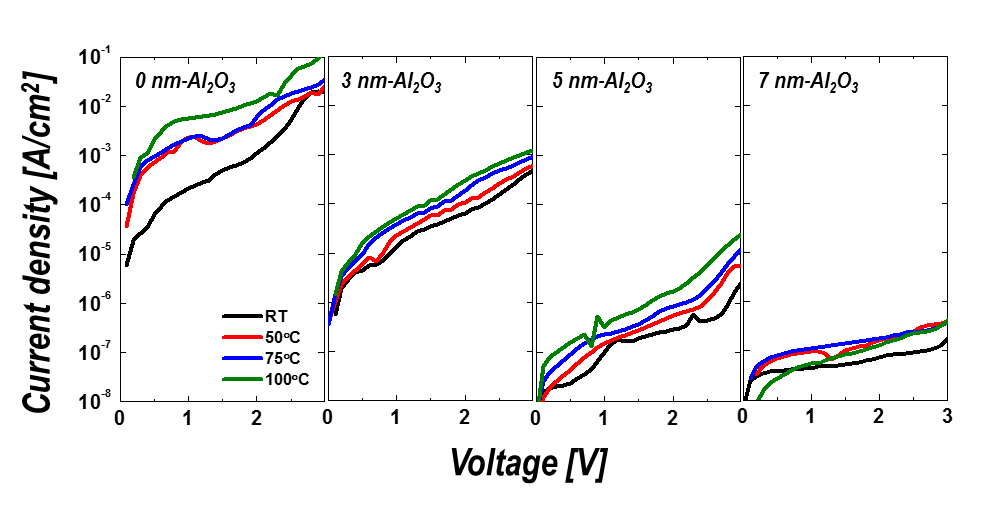


**Figure S2.** *J–V* characteristics of ion gel film with and without Al_2_O_3_ passivation layer measured from room temperature to 100 °C. As the thickness of Al_2_O_3_ layer increases, the current level decreases and the temperature dependence on the leakage current tends to decrease.

**C. AFM analysis of ALD-Al_2_O_3_ layer deposited MoS_2_ crystals.**


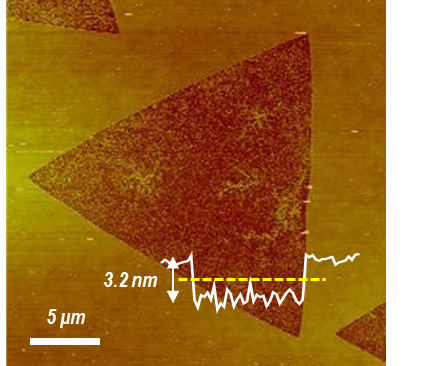


**Figure S3:** AFM image of MoS_2_ crystal after deposition of 3-nm-thick Al_2_O_3_ layer by thermal ALD. Because the ALD process was performed at 350 °C, it was confirmed that the Al_2_O_3_ layer was rarely deposited on the surface of the MoS_2_ crystals, and the Al_2_O_3_ layer was uniformly deposited excepting in the MoS_2_ crystal region.

**References**

1. Li, H. *et al.* From Bulk to Monolayer MoS_2_: Evolution of Raman Scattering. *Adv. Mater.* **22**, 1385–1390 (2012).
